# Supplementary material for: MetaRibo-Seq measures translation in microbiomes
Source: Nat Commun. 2020 Jun 29;11:3268. doi: 10.1038/s41467-020-17081-z (PMC7324362; doi:10.1038/s41467-020-17081-z)
Supplement: Supplementary file 10 — Supplementary Data 7 [file 41467_2020_17081_MOESM10_ESM.zip › File2/Confidence_VeryHigh_Taxonomy/91519_out.krona.html]

Javascript must be enabled to view this page.

members
magnitude
magnitudeUnassigned
count
unassigned
taxon
rank

91519\_out

11

superkingdom
2
11

1239
11
phylum

11
186801
class

order
186802
11

family
3
31979

genus
1485
3


SRS014235\_contig\_number\_24914SRS024435\_contig\_number\_12598
species
1262841
2

1262800
1
species

SRS146812\_contig\_number\_contig-100\_1754.189567

species

SRS019685\_contig\_number\_42206SRS020869\_contig\_number\_17985SRS048870\_contig\_number\_20508SRS052697\_contig\_number\_40707SRS053356\_contig\_number\_contig-100\_3134.260421SRS098571\_contig\_number\_55781SRS1041133\_contig\_number\_22188SRS146812\_contig\_number\_63991
8
1898207
